# Aerobic Vaginitis Diagnosis Criteria Combining Gram Stain with Clinical Features: An Establishment and Prospective Validation Study

**DOI:** 10.3390/diagnostics12010185

**Published:** 2022-01-13

**Authors:** Mengting Dong, Chen Wang, Huiyang Li, Ye Yan, Xiaotong Ma, Huanrong Li, Xingshuo Li, Huihui Wang, Yixuan Zhang, Wenhui Qi, Ke Meng, Wenyan Tian, Yingmei Wang, Aiping Fan, Cha Han, Gilbert G. G. Donders, Fengxia Xue

**Affiliations:** 1 Department of Obstetrics and Gynecology, Tianjin Medical University General Hospital, 154 Anshan Road, He Ping District, Tianjin 300052, China; dongmengting1993@126.com (M.D.); wangchener32@163.com (C.W.); Lihuiyang1992@tmu.edu.cn (H.L.); yanye@tmu.edu.cn (Y.Y.); xiaotongma1992@163.com (X.M.); lihuanrong93@tom.com (H.L.); xingchoco@163.com (X.L.); erwanghuihui@163.com (H.W.); karliezhang9@126.com (Y.Z.); qiwenhui1987@163.com (W.Q.); mengke1104@163.com (K.M.); tianwenyan1108@163.com (W.T.); wangyingmei1978@126.com (Y.W.); fanaipinger@163.com (A.F.); tjhancha@163.com (C.H.); 2 Tianjin Key Laboratory of Female Reproductive Health and Eugenics, 154 Anshan Road, He Ping District, Tianjin 300052, China; 3 Department of Obstetrics and Gynecology, University of Antwerp, 2550 Antwerp-Edegem, Belgium; 4 Femicare Clinical Research for Women, 3300 Tienen, Belgium; 5 Department of Obstetrics and Gynecology, Regional Hospital, 3300 Tienen, Belgium

**Keywords:** aerobic vaginitis, diagnostic criteria, Gram stain, clinical features, 16S rRNA gene sequencing

## Abstract

Wet-mount microscopy aerobic vaginitis (AV) diagnostic criteria need phase-contrast microscopy and keen microscopists, and the preservation of saline smears is less common in clinical practice. This research work developed new AV diagnostic criteria that combine Gram stain with clinical features. We enrolled 325 AV patients and 325 controls as a study population to develop new AV diagnostic criteria. Then, an independent group, which included 500 women, was used as a validation population. AV-related microscopic findings on Gram-stained and wet-mount smears from the same participants were compared. The accuracy of bacterial indicators from the two methods was verified by bacterial 16S rRNA V4 sequencing (*n* = 240). Logistic regression was used to analyse AV-related clinical features. The screened clinical features were combined with Gram-stain microscopic indicators to establish new AV diagnostic criteria. There were no significant differences in the leukocyte counts or the parabasal epitheliocytes (PBC) proportion between the Gram-stain and wet-mount methods (400×). Gram stain (1000×) satisfied the ability to identify bacteria as verified by 16S rRNA sequencing but failed to identify toxic leukocytes. The new criteria included: Lactobacillary grades (LBG) and background flora (Gram stain, 1000×), leukocytes count and PBC proportion (Gram stain, 400×), and clinical features (vaginal pH > 4.5, vagina hyperemia, and yellow discharge). These criteria satisfied the accuracy and reliability for AV diagnosis (Se = 86.79%, Sp = 95.97%, and Kendall’s W value = 0.899) in perspective validation. In summary, we proposed an alternative and valuable AV diagnostic criteria based on the Gram stain, which can make it possible to diagnose common vaginitis like AV, BV, VVC, and mixed infections on the same smear and can be available for artificial intelligence diagnosis in the future.

## 1. Introduction

Aerobic vaginitis (AV) was first proposed in 2002 by Donders et al. [1], which was defined as a vaginal infection primarily with aerobes over lactobacilli, inflammatory reaction, and epithelial atrophy. AV is characterised by yellow vaginal discharge, mucosal hyperaemia, itching, burning, and dyspareunia [2] and accounts for 4.2–25.8% of all vaginal infections [3,4]. Previous studies based on culture, quantitative PCR, and 16S rRNA sequencing indicate the major pathogens of AV include *Escherichia coli*, *Enterococcus faecalis*, *Streptococcus agalactiae*, and *Klebsiella pneumonia* [5,6,7,8,9]. AV is associated with an increased risks of adverse pregnancies [10,11], sexually transmitted infections [12], the persistence of the high risk human papilloma virus, and progression to cervical intraepithelial neoplasia [13]. The IUSTI/WHO incorporated AV into the guidelines in 2018, highlighting the importance of AV diagnosis and treatment [14].

Currently, the most widely used method to diagnose AV is wet-mount microscopy [1,3,14], which needs a phase-contrast microscope (400×) to assess lactobacillary grade (LBG), leukocytes, the proportion of toxic leukocytes, and parabasal epitheliocytes (PBC) and detect background flora (Supplementary Materials Table S1). A composite AV score of ≥3 corresponds to AV. These criteria diagnose AV from the three main characters: LBG and background flora reflect bacteria dysbiosis; the number of leukocytes and the proportion of toxic leukocytes reflect inflammatory reaction, and the proportion of PBC reflect vaginal atrophy. However, several factors limit the broad application of wet-mount microscopy score. First, cells and bacteria can move on saline smears, the long-term preservation of wet-mount smears because repeated review by rehydration is less commonly practiced [15,16]. Second, the identification of aerobes requires a keen microscopist and a phase-contrast microscope, which may be obstacles to wide application in clinical practice in underdeveloped regions and the development of artificial intelligence (AI) interpretation systems. Therefore, it is of great importance to develop new diagnostic methods to complement or compensate for the wet-mount microscopy criteria.

Other AV diagnostic methods include the qPCR method [7,17], bacterial cultivation method [1,2,5,18,19], enzymatic index method [20], and method combining clinical features and microscopic indicators [21]. qPCR and bacterial culture methods can accurately reflect the pathogenic bacteria in AV cases and guide antibiotic treatment. The culture method can also identify antibiotic sensitivity profiles [22], so as to quickly recover and avoid complications caused by the failure of antibiotic treatment. However, the diagnosis of AV is not only based on the presence of aerobic bacteria, but also on variable inflammation and the immaturity of epithelial cells. In the presence of clinical symptoms of inflammation and/or atrophy, qPCR can be reliably substituted for AV diagnosis [7]. Tempera et al. [21] diagnosed AV based on clinical features (yellow vaginal discharge, odour, pH > 5.0) and wet microscopy results (increased leucocytes and LBG), which is a descriptive diagnosis and has not been standardised. Diagnostic tests based on enzymatic indicators include vaginal pH, H_2_O_2_, aerobic bacteria metabolites, inflammatory reaction-related enzymes, and so on with a diagnostic sensitivity of 90%; nevertheless, the enzymatic results are greatly affected by the different kits used and the test environment.

Gram staining is another common diagnostic method for gynaecological infections. Common vaginal infections, such as bacterial vaginosis (BV) [23] and vulvovaginal candidiasis (VVC) [14], have diagnostic criteria based on Gram staining. Gram-stained smears can be magnified 1000 times to identify bacteria under ordinary optical microscopes and can be easily stored for a long time [24]. Moreover, diagnostic methods based on Gram staining make AI diagnosis possible [25]. Whether Gram-stained smears can follow the original wet-mount scoring system to diagnose AV remains to be elucidated. Unlike BV, AV has much more obvious vaginal inflammatory manifestations, such as yellow discharge and vaginal hyperaemia, as well as other markers of host response, such as the presence of parabasal cells. These clinical manifestations can reflect the severity and dynamic changes of AV and are of great importance in guiding the diagnosis and treatment of AV. Therefore, the first aim of this study was to determine whether Gram staining can be used for AV diagnosis by comparing the microscopic examination results of Gram-stained smears and wet-mount smears. The second purpose was to develop AV diagnostic criteria that combined Gram-stained smears and clinical features and to validate the accuracy and reliability of this new criteria in a prospective population.

## 2. Materials and Methods

### 2.1. Study Design and Participants

A total of 1150 participants were enrolled from December 2014 to August 2020, including the study population (*n* = 650) and validation population (*n* = 500). The study population (*n* = 650) comprising 325 AV patients enrolled from the gynaecology outpatient department and 325 healthy controls from the health management centre at Tianjin Medical University General Hospital from December 2014 to September 2019.

For study population, microscopic findings were compared between wet-mount and Gram-stained smears. Among them, vaginal bacteria 16S ribosomal RNA gene sequencing was conducted in 80 AV patients and 160 controls. The sequencing results were used as a benchmark to evaluate the accuracy of the two methods for assessing LBG and background flora. Finally, the AV-associated clinical features that can be included in the diagnostic criteria were screened, and new criteria that combined Gram-stained smears with clinical features were established.

**Figure 1:**
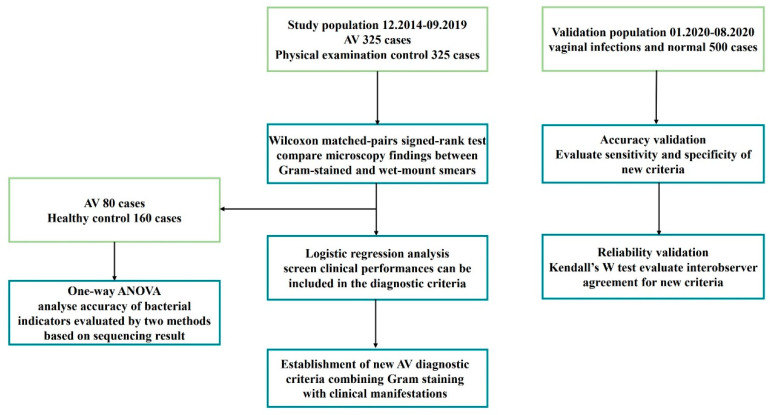
Flowchart of establishment of new AV diagnostic criteria.

The validation population consisted of 500 consecutive participants who received vaginal discharge examinations in the gynaecology outpatient department from January 2020 to August 2020. The accuracy and reliability of the new criteria were validated (Figure 1).

### 2.2. Inclusion and Exclusion Criteria

For study population, the inclusion criteria were as follows: (1) women of reproductive age; (2) with regular menstruation; (3) had sexual intercourse history; (4) women who had an AV score ≥3 according to wet-mount microscopy were enrolled as experimental group. Healthy controls were selected from women who presented for routine examinations at health management centre in the corresponding period. The exclusion criteria include: (1) pregnant, lactating, menopausal, or during the menstrual period; (2) suffered from other vaginitis (BV, VVC, and TV); (3) suffered from cervicitis and pelvic inflammatory diseases; (4) engaged in sexual intercourse or vaginal douching within 3 days; (5) used antibacterial (local or systemic) therapy within 1 week.

For validation population, the inclusion criteria were as follows: (1) women of reproductive age; (2) had sexual intercourse history; (3) consecutive cases who received vaginal discharge examinations in the gynaecology outpatient department. The exclusion criteria include (1) pregnant, lactating, menopausal, or during the menstrual period; (2) suffered from cervicitis and pelvic inflammatory diseases; (3) engaged in sexual intercourse or vaginal douching within 3 days; (4) used antibacterial (local or systemic) therapy within 1 week. Written informed consent was obtained from all participants.

### 2.3. Clinical History and Sample Collection

Each participant completed a standard questionnaire containing demographic information, history of pregnancy and menstruation, and medical history. Symptoms, such as vaginal discharge, vaginal dyspareunia, vulvovaginal burning, and itching, were recorded. All participants received vaginal examination for hyperaemia, discharge (colour, consistency, and volume), cervical congestion, and purulent discharge.

Vaginal samples were collected from each participant using 3 sterile cotton sticks (from the lateral upper vaginal wall) for wet-mount smears and Gram-stained smears and vaginal pH. A pH > 4.5 was considered pathological. Then, vaginal lavage fluid was collected from 80 AV cases and 160 controls to sequence the vaginal bacterial 16S rRNA V4 region. Vaginal lavage was prepared by rinsing vaginal wall with 5 mL of sterile 0.9% NaCl solution using a sterile syringe.

### 2.4. Vaginal Smear Examination

Wet-mount smears were prepared using previously described methods [26]. Briefly, a sample of vaginal secretions was suspended in 0.5 mL normal saline, and appropriate amounts of suspension were transferred to a slide, covered with a slip, and examined under a phase-contrast microscope (OLYMPUS; Japan) at 400× magnification. Gram-stained smears were prepared according to the manufacturer’s instructions (Zhuhai Beisuo Biological Technology Co., Ltd., Zhuhai, China). The Gram-stained smears were viewed at both 400 and 1000 magnifications.

Both wet-mount and Gram-stained smears were examined by 3 designated observers independently and blindly. Microscopy evaluations were based on at least 10 view fields randomly selected from each smear. For LBG and background flora, the results were considered final if 2 or more observers reached the same conclusion. The mean leukocyte counts and proportion of PBC from the 3 observers were recorded. The AV score of wet-mount smears was finally calculated on the basis of the results from all 3 observers.

DNA from vaginal lavage was extracted, and PCR amplification and sequencing targeting the V4 regions of the 16S rRNA gene were performed on a HiSeq2500 (Illumina, San Diego, CA, USA). Bioinformatics analysis and results were already reported in a previous study [15].

### 2.5. Diagnostic Criteria

AV was diagnosed if the composite score was ≥3 based on the wet-mount smear diagnostic criteria [3,14]. LBG were defined by Donders et al. [1]: LBG grade I flora correspond predominantly to lactobacillary morphological types, IIa predominantly to lactobacilli but mixed with other bacteria, Iib predominantly to other bacteria overgrowth but limited numbers of lactobacilli are still present, and III predominantly to microflora consisting of numerous other bacteria, with no lactobacilli present. The BV and VVC were diagnosed using a Nugent score of ≥7 [23], and the presence of blastospores and pseudohyphae or hyphae [14] on Gram-stained smears, respectively. Trichomonas vaginitis (TV) was diagnosed by fresh wet-mount microscopy [23]. Vaginal microenvironment disturbance was diagnosed if the vaginal dominant bacteria are abnormal, and the leukocyte count was increased without evidence of other vaginal infections.

### 2.6. Statistical Analysis

All data were analysed by SPSS V22.0. Normally distributed measurement data are presented as the means ± standard deviation (SD). Data that did not follow a normal distribution are presented as medians ± quartiles. Enumeration data are presented as numbers (percentages). The Wilcoxon matched-pairs signed-rank test was used to compare pair-designed non-normally distributed data. A nonparametric test was used to compare multigroup non-normally distributed measurement data. Logistic regression analysis was used to select clinical features common to AV. ROC curve analysis was used to determine the cut-off value for AV diagnosis. PASS 15.0 statistical software was used to calculate the sample size of the validation population. The sensitivity, specificity, and Youden index were calculated to evaluate the accuracy of the new criteria. Kendall’s W test was adopted to measure the interagreement of microscopic findings. A *p* value < 0.05 was considered statistically significant.

## 3. Results

### 3.1. General Characteristics of the Participants

A total of 325 AV and 325 controls were enrolled as the study population. The age of the AV group ranged from 20 years to 55 years (mean 34.83 ± 9.23 years) with a mean of 32.99 years. The age of the controls ranged from 18 years to 50 years with a mean of 31.78 years. There was no significant difference between the average age of the AV group and the control group (32.99 ± 7.45 vs. 31.78 ± 7.14, *p* > 0.05). Since the minimum sample size of the theoretical validation population was 492, 500 participants were enrolled as the validation population with ages ranging from 16 years to 54 years (mean 34.83 ± 9.23 years). The demographic and clinical information of the participants is presented in Table S2.

### 3.2. Diagnostic Performance of Gram Stain

#### 3.2.1. Comparison of Microscopic Findings from Gram-Stained and Wet-Mount Smears

Pairwise comparison showed no statistical significance in leukocyte counts, leukocyte/epithelial cell ratio, or PBC proportion between the wet-mount and Gram-stained smears at 400× magnification (Table 1). Both LBG and background flora showed no difference between wet-mount smears at 400× and Gram-stained smears at 1000× magnification (Table 1). While identifiable on a wet-mount smear, toxic leukocytes were indistinguishable under Gram-stained smears. According to the above results, we chose 400× magnification to assess leukocyte counts and PBC proportions and 1000× magnification to assess LBG and background flora. Microscopic findings of AV from wet-mount and Gram-stained smears are shown in Figures S1–S5.

#### 3.2.2. Correlation between Bacterial Indicators Evaluated by Two Methods with 16S rRNA Sequencing

The bacterial species with a relative abundance of ≥0.1% in 240 vaginal samples were categorised into lactobacillus-like, enterobacteria-like, and cocci-like floras according to their morphologies [27].

**Figure 2:**
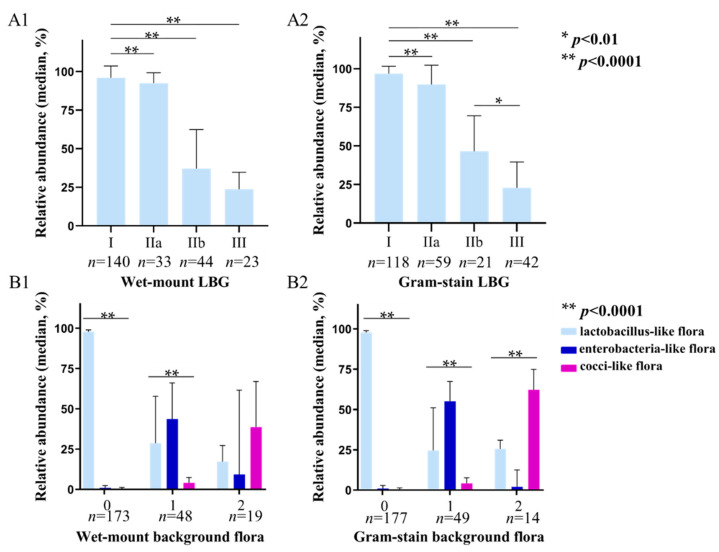
16S rRNA sequencing results according to LBG and background flora group evaluated by wet-mount (400×, phase-contrast microscope) and Gram-stained smears (1000×, oil lens). (**A1**,**A2**) show the average relative abundance of lactobacilli in each group of LBG I, IIa, IIb, and III by wet mount and Gram stain microscopy, respectively; (**B1**,**B2**) show the average relative abundance of lactobacilli, enterobacteria-like bacteria, and cocci-like bacteria in background flora group 0, 1, and 2 by wet-mount and Gram stain microscopy. *: *p* < 0.01, **: *p* < 0.0001.

We evaluated the accuracy of LBG determined by wet-mount (400×, phase-contrast microscope) and Gram-stained (1000×, oil lens) smears (Figure 2, Figures S1 and S2). For this, 240 samples were divided into four groups according to the LBG results determined by wet-mount and Gram-stained smears, respectively. The results show that the average relative abundance of lactobacilli gradually decreased in both the wet-mount group and Gram-stain group. All the differences between each Gram-stain LBG group were statistically significant (*p* < 0.0001). In the wet-mount group, only the difference between LBG IIb and LBG III was not significant (*p* = 0.155).

The correlation between background flora evaluated by wet-mount and Gram-stained smears with sequencing results is shown in Figure 2(B1,B2). According to the background flora determined by wet-mount smears, 240 subjects were divided into group 0 (no other bacteria), group 1 (small coliform bacilli), and group 2 (cocci or chains). The average relative abundance of lactobacilli and enterobacteria-like bacteria was the highest in group 0 and group 1, respectively (*p* < 0.0001, *p* < 0.0001), but in group 2, the average relative abundance of cocci-like bacteria was not significantly higher than that of the other two forms of bacteria (*p* = 0.263). In the Gram stain group, the average relative abundance of lactobacilli in group 0 (*p* < 0.0001) and enterobacteria-like bacteria in group 1 (*p* < 0.0001) were also the highest. Moreover, the average relative abundance of cocci-like bacteria in group 2 was the highest (*p* < 0.0001), which was significantly higher than that of lactobacilli and enterobacteria-like bacteria (*p* = 0.007, *p* < 0.0001).

### 3.3. New AV Diagnostic Criteria

#### 3.3.1. Logistic Analysis for AV Clinical Features

We used logistic regression analysis to identify specific clinical features to substitute toxic leukocytes that cannot be assessed in Gram-stained smears (Table 2). Among the seven entries, vaginal hyperaemia, yellow discharge, and elevated vaginal pH were closely associated with AV and therefore chosen to be included in the new criteria.

#### 3.3.2. Establishment of New AV Diagnostic Criteria

To facilitate the clinical application of the new criteria, the score of the above four Gram-stained microscopic indicators still referenced the original wet-mount microscopy score system. To establish new criteria, the above four microscopic findings were incorporated with three clinical features in different permutation combinations. The sensitivity, specificity, and Youden index of the AV diagnosis were calculated (Table S3). The combination with the highest accuracy was incorporated into the new criteria. Zero corresponds to normal clinical features, 1 to either elevated vaginal pH or at least one abnormal signs, and 2 to both high vaginal pH and at least one abnormal signs, where abnormal signs include vaginal hyperaemia and yellow discharge.

The new criteria were therefore developed as follows: leukocyte counts and PBC proportion at 400× magnifications, LBG and background flora at 1000× magnifications, and key clinical features (vaginal pH, vaginal hyperaemia, and yellow discharge) (Table 3). According to the ROC curve analysis, AV can be diagnosed if the composite score is 4 or higher; mild, moderate, and severe AV can be diagnosed if the composite score is 4–5, 6–7, and 8–10, respectively (Figure S6). When compared with the wet-mount microscopy criteria, the new criteria achieved 96.92% sensitivity and 97.54% specificity for AV diagnosis (Table S4). The total accuracy for the diagnosis of normal, mild, moderate, and severe AV was 84.92% (552/650).

### 3.4. Prospective Validation of New AV Diagnostic Criteria

#### 3.4.1. Accuracy of the New AV Diagnostic Criteria

We prospectively verified the new AV diagnosis criteria in an independent general population consisting of 298 normal and vaginal microenvironment disturbance participants and 202 vaginal infection patients, which included 53 simple AV cases diagnosed by wet-mount microscopy. Fifty BV cases, 29 VVC cases, and 9 BV + VVC cases were diagnosed by Gram staining. Seventy mixed vaginal infections, which included 60 AV mixed infections (36 AV + BV, 18 AV + VVC, 6 AV + BV + VVC) and 1 BV + TV case were diagnosed by Gram staining combined with wet-mount microscopy.

The new diagnostic criteria have satisfied accuracy for simple AV diagnosis with a sensitivity of 86.79% (46/53) and a specificity of 95.97% (286/298). Meanwhile, BV and VVC can be simultaneously diagnosed by Gram-stained smears; the sensitivity of AV and its mixed infection was 82.30% (93/113), and the specificity was 94.83% (367/387) (Table 4).

The inconsistent diagnostic results of the two criteria are as follows (Table S5): Among 113 simple and mixed AV cases who met wet-mount AV diagnosis, 20 cases were not diagnosed by the new criteria, 18 of which were mild AV. Among the above 18 cases, 12 were asymptomatic mild AV.

Among 298 normal and vaginal microenvironment disturbance patients, 12 could be diagnosed with AV using the new criteria, and eight patients had abnormal clinical features.

In addition, 50 cases of simple BV, 29 cases of simple VVC, 9 cases of BV + VVC, and 1 case of BV + TV could be accurately diagnosed on Gram-stained smears. Among them, eight cases were diagnosed with AV by new criteria (three BV cases with vaginal mucosal hyperaemia and two VVC cases with elevated pH), suggesting that Gram staining can be used to diagnose multiple types of vaginal infections and mixed vaginal infections.

#### 3.4.2. Interobserver Agreement of the New AV Diagnostic Criteria

For reliability, we used Kendall’s W test which was suitable for three or more observers to test interobserver agreements using the two criteria (Table 5). The interobserver agreement for LBG and background flora determined by Gram staining was slightly higher than wet-mount smears (Kendall’s W = 0.876 vs. Kendall’s W = 0.828; 0.713 vs. 0.603). Meanwhile, the interobserver agreement for leukocyte counts and PBC proportion determined by the two methods was comparable (0.778 vs. 0.771; 0.544 vs. 0.544). In addition, the interobserver agreement of the new criteria in the evaluation of AV scores was satisfied (KW = 0.899).

## 4. Discussion

Since AV was proposed, the diagnostic method has attracted much attention worldwide [28,29,30]. Gram stain is the basic bacterial identification method [31] and is widely used in the diagnosis of some common vaginal infections, such as BV and VVC. Although some studies proposed that Gram stain can be used to confirm vaginal infections and background flora [29,32], the precise role of Gram stain in diagnosing AV has still not been unequivocally demonstrated. This study represented the first clinical study to provide evidence that Gram stain can be used to evaluate the AV microscopy indicators by comparing Gram-stained and wet-mount microscopy findings and further evaluating the correlations with vaginal sequencing results. Subsequently, we proposed new AV diagnostic criteria combining Gram-stained smear microscopy with clinical features which proved to have satisfied diagnostic accuracy and bacteria identification ability.

We firstly compared the difference between Gram-stained and wet-mount smears from the same woman. Regarding the number of leukocytes and the proportion of PBC, the differences between Gram stained and wet-mount smears at the same magnification (400×) were not statistically significant. In addition, the interobserver agreements between the two methods were also comparable (0.778 vs. 0.771; 0.544 vs. 0.544). Although some reports considered that the presence of leukocytes and changes in epithelial cells are not well-addressed in Gram-stained smears [28], our research indicates there is no significant difference in the evaluation and reliability of the cell parameters between the two methods. However, Gram-stained smears fail to observe toxic leukocytes, because the toxic granules were undistinguishable after the procession of heat fixation and staining.

As for LBG and background flora, several studies from Donders et al. also compare the LBG results between Gram stain and wet mount [33,34,35]. In their study, they air dried smears, and Gram stains were performed within a maximum of six hours. Their results show that the LBG evaluated by Gram stain was higher than wet-mount, and the wet-mount results correlated better with lactic acid [34]. In our study, Gram stain and wet mount were performed simultaneously and immediately after the vaginal discharge were taken during speculum examination. Nevertheless, we also found that the LBG from Gram stain are more likely higher than wet mount in general (Table 1, Figure 2), even though the difference between wet-mount (400×) and Gram-stained smears (1000×) were not significant when compared the paired smear results from the same women. In our study, Gram stain is more likely to determine wet-mount LBG I as LBG IIa, and wet-mount LBG IIb as LBG III. This finding was noted in earlier work and maybe due to the fact that some lactobacilli were lost during the procession of Gram staining [33]. However, our sequencing results show that the relative abundance of lactobacilli in Gram-stained LBG IIa and III were slightly lower than wet-mount (95.32%, 86.13%/98.46% vs. 95.32% 88.65%/98.05%; 18.21%, 11.15%/25.93% vs. 22.59%, 18.19%/25.85%), indicating that more cases with lower lactobacillus abundance were determined by a higher LBG by Gram stain. In the lactobacillus dominant group, Gram stain is more likely to find small abnormal bacteria on smears at 1000 magnification. In the abnormal bacteria dominant group, Gram stain can differentiate rod-shaped nonlactobacilli and lactobacilli with the guidance of staining status. Intriguingly, we found the average abundance of lactobacilli was approximately 20% in both the Gram stain and wet mount LBG III groups, which are defined as no lactobacilli present on the smears, and *Lactobacillus iners* were the most prevalent lactobacillus in LBG III group. Some research proved that *Lactobacillus iners* had Gram-negative staining appearances, and were hardly distinguishable from other Gram-negative enterobacilli under microscopy [36].

Meanwhile, the sequencing results in microscopy background flora groups also showed that the abundance of cocci-like flora was significantly highest in the Gram stain group 2. The abundance of enterobacteria-like and cocci-like bacteria in the Gram stain group 1 and group 2 were also higher than the wet-mount group 1 and group 2, respectively. Gram stain can distinguish Gram-negative enterobacteria from mainly Gram-positive cocci from both morphology and staining. Such distinction in wet mounts is purely morphological. The slightly higher reliability of LBG and background flora by Gram stain (Table 5) also demonstrated the good ability in bacteria identification.

Subsequently, we selected and incorporated three clinical manifestations with Gram stain microscopy findings. On the one hand, the clinical features can substitute toxic leukocytes, which cannot be recognised under Gram stain. More importantly, clinical features are of great guiding value in AV diagnosis and treatment. Therefore, we choose three clinical features, which were most associated with AV incorporated into the new Gram stain criteria. The new criteria were proven to be of satisfying diagnostic performance in the validation population. The sensitivity and specificity of the new criteria for diagnosing simple AV were 86.79% (46/53) and 95.97% (286/298). By analysing the patients with inconsistent diagnostic results, we found that among the 20 AV patients who met wet-mount criteria but did not meet the new criteria, 12 were mild AV without clinical features; 20 patients met the new criteria but did not fulfil wet-mount criteria, and 12 patients had abnormal AV-related clinical features. It can be speculated that some asymptomatic mild AV may be a vaginal disturbance interim state, and immediate intervention may be unnecessary. For those vaginal disturbance diagnosed by wet-mount criteria but with typical clinical features, the possibility of AV cannot be completely ignored.

Multiple infections can account for as many as 56.8% of vaginal infections [37] and are a frequent cause of treatment failure. AV is more likely to be combined with other infections [2]. Currently, BV [23] and VVC [14] have corresponding diagnostic criteria based on Gram-stained smears. Applying this Gram-stain-based diagnostic criteria, the above-mentioned three common types of vaginal infection can be diagnosed on the same Gram-stained smear simultaneously, which improves the diagnostic efficiency and has good feasibility and generalizability. Recently, AI diagnosis of infectious diseases based on Gram stain has become an emerging interdisciplinary technology [25,38]. The proposal and application of these diagnostic criteria will lay a foundation for developing AV artificial intelligence diagnostic models.

In this study, we developed new AV diagnostic criteria combining Gram stain and clinical features. These new criteria showed satisfactory diagnostic accuracy and bacteria identification ability when using wet mount as the gold standard. The wet-mount score system is suitable for the areas with phase-contrast microscopy and keen microscopists. This Gram-stain-based diagnostic criteria can be applied by traditional ordinal light microscopy, which is suitable for undeveloped areas lacking phase-contrast microscopes. There are some limitations to our new approach. Firstly, Gram stain fails to evaluate toxic leukocytes. Toxic leukocytes reflect the inflammatory character of AV. The three clinical features we selected included elevated pH value, vaginal hyperaemia, and yellow discharge and also reflected vaginal inflammation reaction which can compensate for or substitute the effect of toxic leukocytes. Secondly, it was composed of 400× 1000× microscopy and clinical data. On the one hand, the new criteria can diagnose AV from clinical and laboratory features comprehensively. On the other hand, it is more complicated in routine practice and relies on the proper use of microscopy and correct clinical data. Another limitation is about the time getting results, unlike wet-mount smears, which allow direct application when patients are still in the consulting room, patients need to wait longer for Gram-stained results because the application needs more time.

## 5. Conclusions

In summary, our study developed alternative and valuable new AV diagnostic criteria that combine Gram staining and clinical features with satisfactory accuracy and bacteria identification ability. The proposed diagnostic criteria make it possible to diagnose multiple vaginal infections on the same Gram-stained smear. and lay the foundation for the development of an AV artificial intelligence diagnostic model.

## Figures and Tables

**Table 1:** Comparison of Gram-stained smears and wet-mount smears in evaluation of microscopy findings.

AV Related Microscopic Findings	Wet-Mount Smear (400×) *n* (%)	Gram-Stained Smear (400×) *n* (%)	Z *^,#^	p *^,^ ^#^	Wet-Mount Smear(400×) *n* (%)	Gram-Stained Smear (1000×) *n* (%)	Z **^,#^	*p* **^,#^
Leukocyte counts	11.8 (3.20, 26.95)	12.0 (2.83, 28.97)	−1.43	0.15				
Toxic leukocyte/ Leukocyte ratio	0.00% (0.00%, 33.83%)	-	-	-				
PBC ratio	0.00% (0.00%, 0.00%)	0.00% (0.00%, 0.00%)	−0.15	0.88				
LBG score			−2.23	0.03			−1.60	0.11
0 (I, IIa)	282 (43.38)	293 (45.08)			282 (43.38)	288 (44.31)		
1 (IIb)	145 (22.31)	58 (8.92)			145 (22.31)	83 (12.77)		
2 (III)	223 (34.31)	299 (46.00)			223 (34.31)	279 (42.92)		
Background flora score			−2.05	0.04			−1.19	0.23
0 (No other bacteria)	316 (48.62)	326 (50.15)			316 (48.62)	327 (50.31)		
1 (Small coliform bacilli)	254 (39.08)	278 (42.77)			254 (39.08)	262 (40.31)		
2 (Cocci or chains)	80 (12.31)	46 (7.08)			80 (12.31)	61 (9.38)		

Parabasal epitheliocytes (PBC), Lactobacillary grades (LBG) *: wet-mount smear (400×, phase-contrast microscope) vs. Gram-stained smear (400×, optical microscope). **: wet-mount smear (400×, phase-contrast microscope) vs. Gram-stained smear (1000×, oil lens). ^#^: Non-normally distributed data are compared using Wilcoxon matched-pairs signed-rank test.

**Table 2:** Logistic regression analysis of AV clinical manifestations.

Clinical Manifestations	Univariate Logistic Regression Analysis		Multivariate Logistic Regression Analysis	
	*p*	OR (95% CI)	*p*	OR (95% CI)
Vulvovaginal itching				
no				
yes	<0.0001	4.651 (3.040−7.143)	0.066	2.091 (0.952−4.593)
Vaginal dyspareunia				
no				
yes	0.001	11.905 (4.219−33.333)	0.153	3.388 (0.635−18.072)
Increased vaginal discharge				
no				
yes	<0.0001	5.348 (3.817−7.519)	0.064	1.878 (0.964−3.662)
Yellow discharge				
no				
yes	<0.0001	26.316 (11.364−62.500)	<0.0001	10.189 (2.907−35.714)
Vaginal hyperemia				
no				
yes	<0.0001	16.129 (9.901−26.316)	<0.0001	5.092 (2.269−11.427)
pH value				
≤4.5				
>4.5	<0.0001	14.925 (10.204−22.222)	<0.0001	6.542 (3.421−12.509)

**Table 3:** Diagnostic criteria for AV Gram staining combined with clinical manifestations *.

Score	LBG (1000×)	No. of Leukocytes (400×)	Background Flora (1000×)	Proportion of PBC (400×)	Clinical Manifestation
0	I, IIa	≤10/hpf	No other bacteria	<1%	pH ≤ 4.5 and no abnormal signs ^#^
1	IIb	>10/hpf and ≤10/epithelial cells	Small coliform bacilli	≥1% and ≤10%	pH > 4.5 or at least one of abnormal signs ^#^
2	III	>10/epithelial cells	Cocci or chains	>10%	pH > 4.5 and at least one of abnormal signs ^#^

* The number of leukocytes and the proportion of parabasal epitheliocytes (PBC) were evaluated by light microscopy (400× magnification). Lactobacillary grades (LBG) and background flora were evaluated by oil immersion (1000× magnification). ^#^ Abnormal signs include vaginal hyperaemia and yellow discharge. hpf: high power field. A composite AV score of <4 corresponded to normal, 4~5 to ‘light AV’, 6 to 7 to ‘moderate AV’, and >7 to ‘severe AV’.

**Table 4:** Accuracy validation of new diagnostic criteria.

New Criteria AV	Wet-Mount Simple AV		Total	Sensitivity (%)	Specificity (%)	Youden Index	Wet-Mount Simple and Mixed AV		Total	Sensitivity (%)	Specificity (%)	Youden Index
	Yes	No					Yes	No				
Yes	46	12	58	86.79	95.97	0.828	93	20	113	82.30	94.83	0.771
No	7	286	293				20	367	387			
Total	53	298	351				113	387	500			

**Table 5:** The comparison of interobserver agreement between the two criteria.

Indicators	New Diagnostic Criteria KW Value	Wet-Mount Diagnostic Criteria KW Value
LBG	0.876	0.828
Background flora	0.713	0.603
No. of leukocytes	0.778	0.771
PBC proportion	0.544	0.544
AV score	0.899	0.811

## Data Availability

The authors declare that the data of this study are available from the corresponding author on reasonable request.

## Supplementary Materials

The following are available online at https://www.mdpi.com/article/10.3390/diagnostics12010185/s1. Table S1: AV wet-mount microscopy diagnostic score. Table S2: The characteristics of participants. Table S3: The accuracy of different clinical features combinations. Table S4: Diagnostic performance of new criteria for diagnosing AV. Table S5: The inconsistent diagnostic results of the two criteria in validation study. Figure S1: Comparison of LBG microscopic findings between Gram-stain (400×; 1000×) and wet-mount smears (400×). Figure S2: Comparison of background flora between Gram-stained (400×; 1000×) and wet-mount smears (400×). Figure S3: Comparison of leukocyte count between Gram-stained (400×; 1000×) and wet-mount smears (400×). Figure S4: Comparison of PBC proportion between Gram-stained (400×; 1000×) and wet-mount smears (400×). Figure S5: Comparison of proportion of toxic leukocytes between Gram-stained (400×; 1000×) and wet-mount smears (400×). Figure S6: The ROC curve for the diagnosis of AV with different levels of severity with new diagnostic criteria.
