# Supplementary material for: Aerobic Vaginitis Diagnosis Criteria Combining Gram Stain with Clinical Features: An Establishment and Prospective Validation Study
Source: Diagnostics (Basel). 2022 Jan 13;12(1):185. doi: 10.3390/diagnostics12010185 (PMC8775230; doi:10.3390/diagnostics12010185)
Supplement: Supplementary file 1 [file diagnostics-12-00185-s001.zip › supplementary figures S1-S6.pdf]

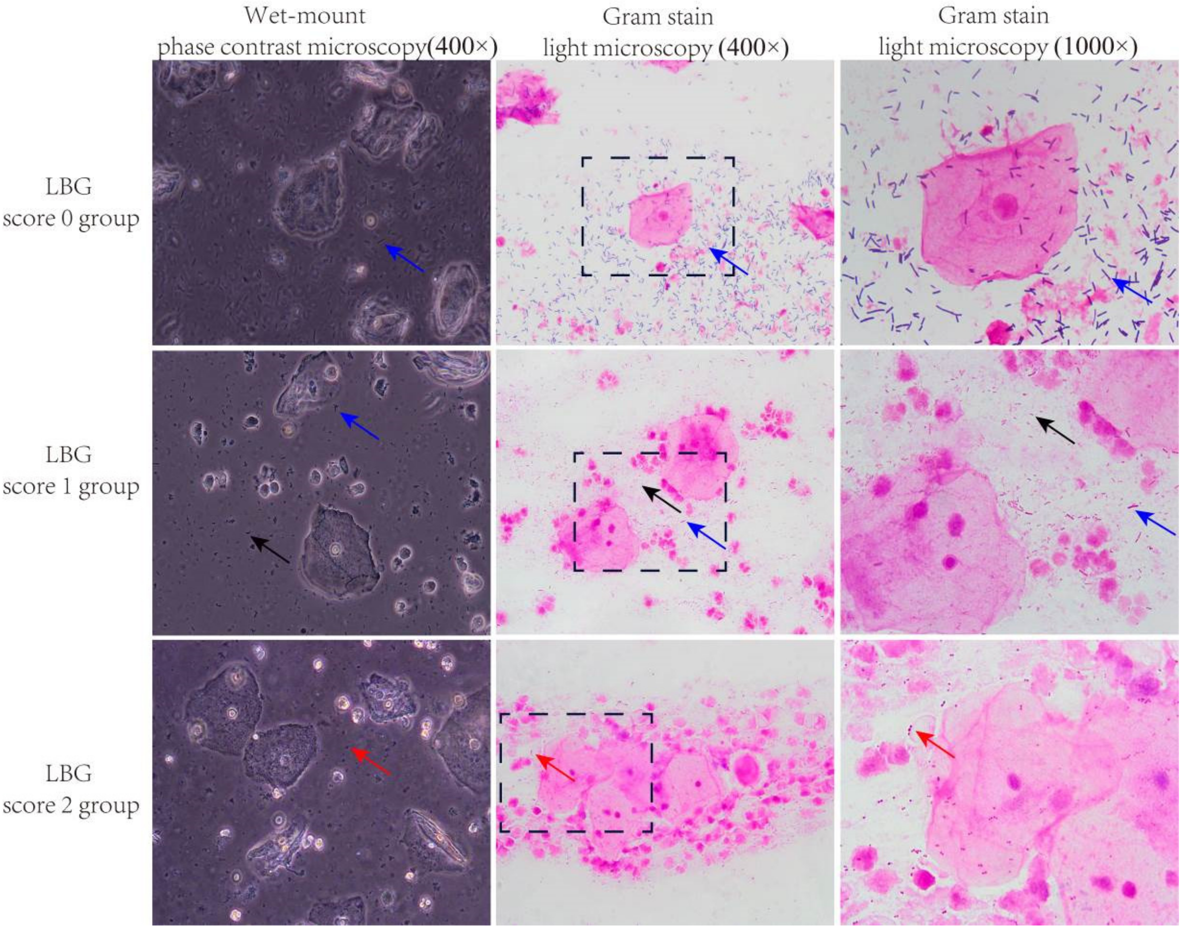

Figure S1. Comparison of LBG microscopic findings between Gram stain smear (400×; 1000×) and wet mount (400×).

Pictures in the same row from left to right displayed microscopic images at wet mount (400×; phase contrast microscope), Gram stain (1000×; optical microscope) and Gram stain (1000×; oil lens) from the same participant. Pictures in the same column from top to bottom showed microscopic images in according to LBG score 0,1 and 2 from three different participants. The blue arrows indicated lactobacilli-like bacteria, the black arrows indicated enterobacteria-like bacteria, and the red arrows indicated cocci-like bacteria. Black dotted box area at Gram stain (400 ×) was further magnified to Gram stain (1000 ×), Lactobacilli-like bacteria appeared as Gram-positive bacilli at Gram stain.

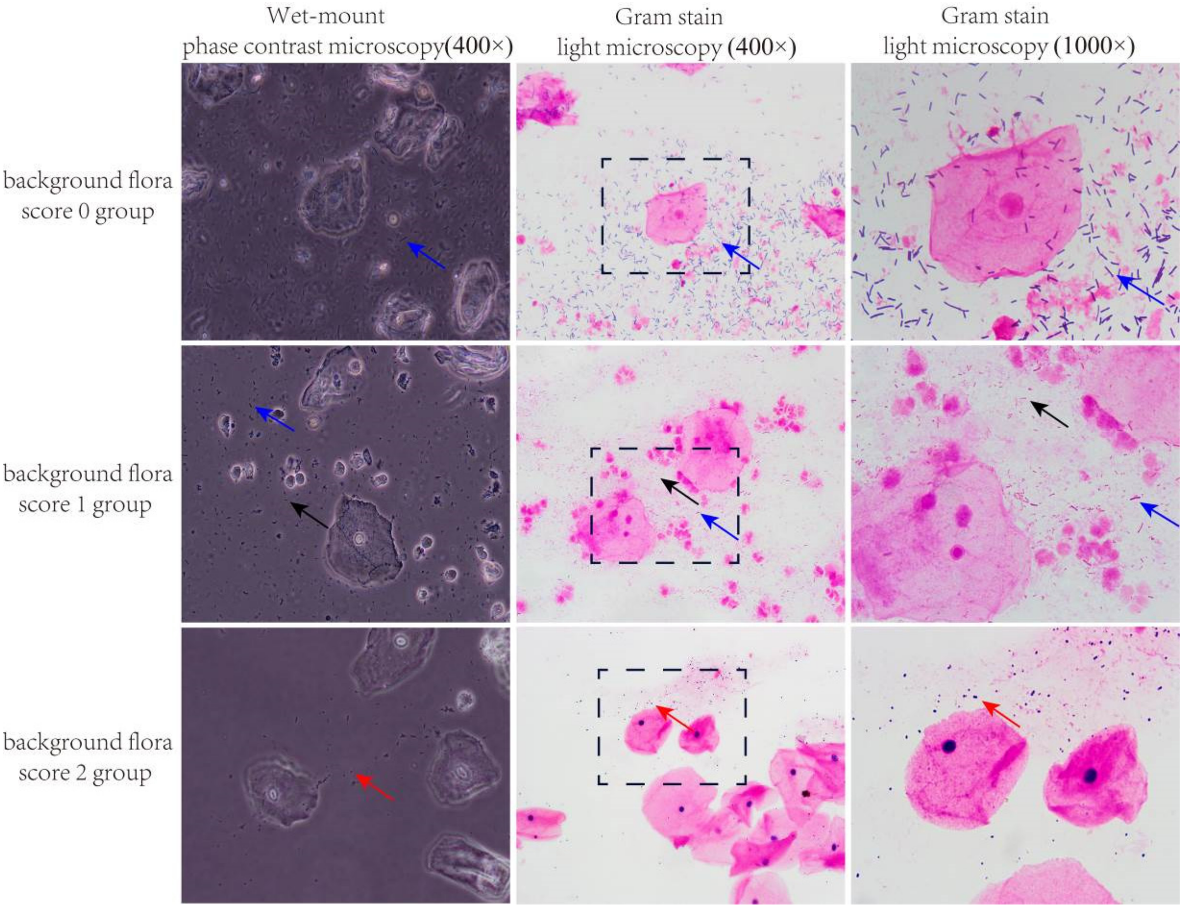

Figure S2. Comparison of background flora between Gram-stained (400×; 1000×) and wet-mount smears (400×). Pictures in the same row from left to right displayed microscopic images at wet mount (400×; phase contrast microscope), Gram stain (1000×; optical microscope) and Gram stain (1000×; oil lens) from the same participant. Pictures in the same column from top to bottom showed microscopic images in according to background flora score 0,1 and 2 from three different participants. The blue arrows indicated lactobacilli-like bacteria, the black arrows indicated enterobacteria-like bacteria, and the red arrows indicated cocci-like bacteria. At Gram stain, Lactobacilli-like, enterobacteria-like and cocci-like bacteria appeared as Gram-positive bacilli, small Gram-negative bacilli and Gram-positive cocci (monococci, diplococci or chains-like), respectively. Black dotted box area at Gram stain (400 ×) was further magnified to Gram stain (1000 ×), which showed the morphology and proportion of above different background flora were clearer than those at wet mount (400×).

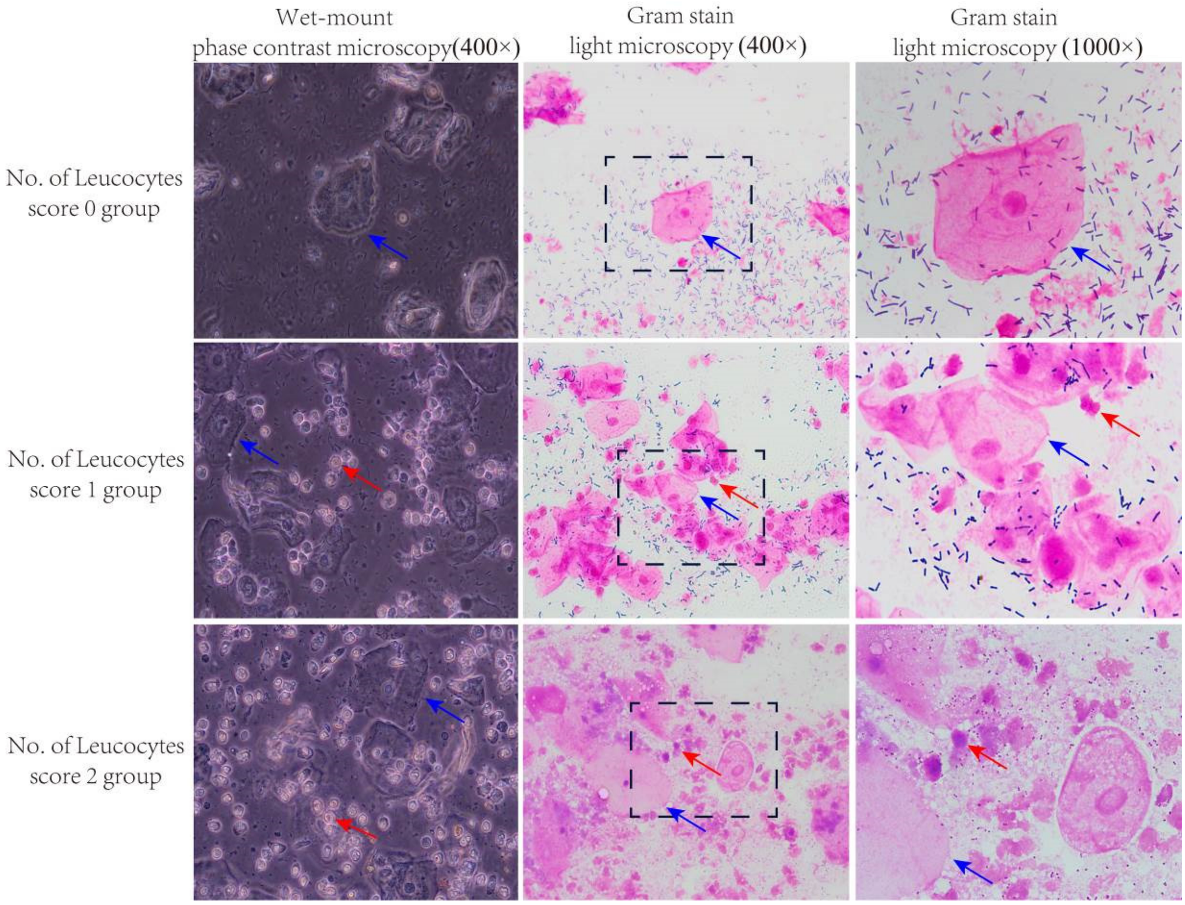

Figure S3. Comparison of leucocyte count between Gram-stained (400×; 1000×) and wet-mount smears (400×). Pictures in the same row from left to right displayed microscopic images at wet mount (400×; phase contrast microscope), Gram stain (1000×; optical microscope) and Gram stain (1000×; oil lens) from the same participant. Pictures in the same column from top to bottom showed microscopic images in according to leucocyte count score 0,1 and 2 from three different participants. The blue arrows indicated vaginal epithelial cells, and the red arrows indicated leucocytes. Black dotted box area at Gram stain (400 ×) was further magnified to Gram stain (1000 ×), which showed the number of countable leucocytes decreased.

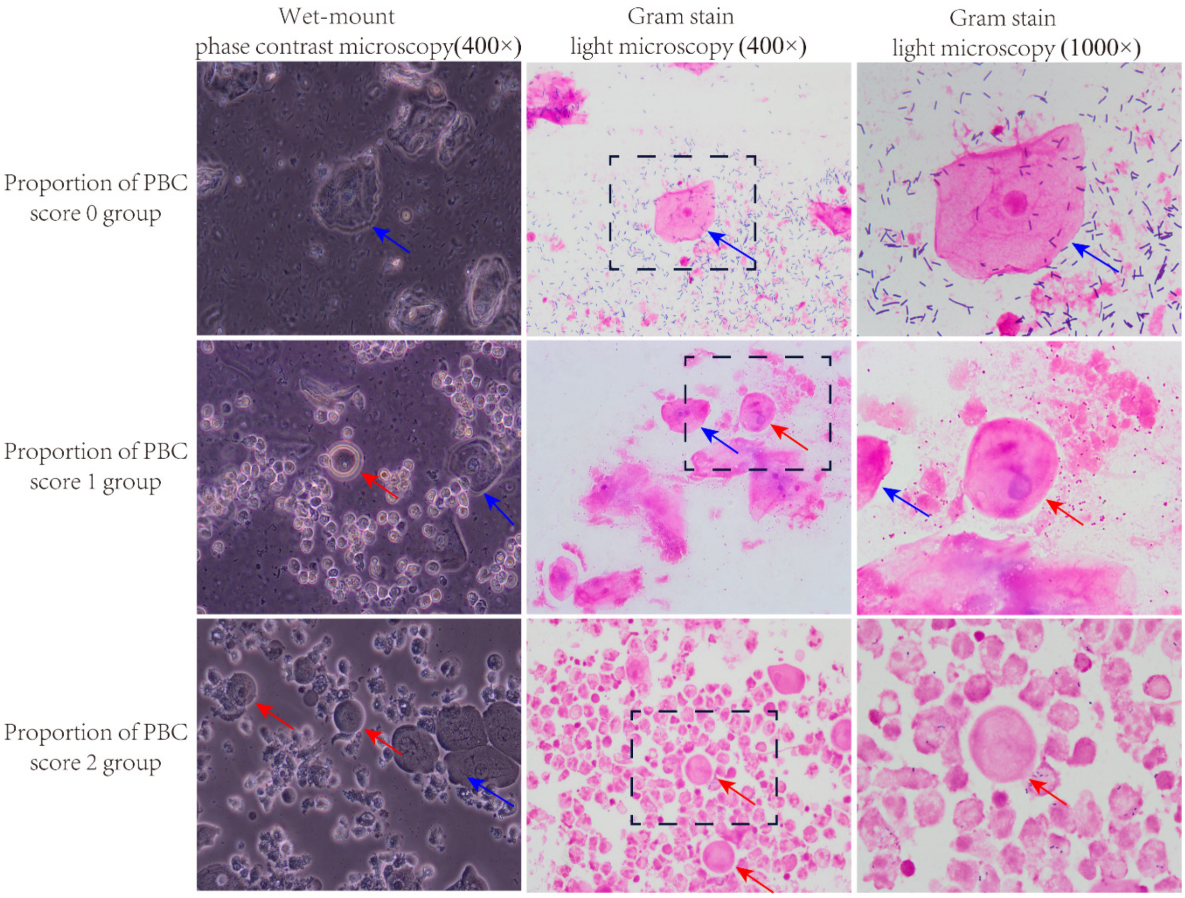

Figure S4. Comparison of PBC proportion between Gram-stained (400×; 1000×) and wet-mount smears (400×). Pictures in the same row from left to right displayed microscopic images at wet mount (400×; phase contrast microscope), Gram stain (1000×; optical microscope) and Gram stain (1000×; oil lens) from the same participant. Pictures in the same column from top to bottom showed microscopic images in according to of PBC proportion score 0,1 and 2 from three different participants. The blue arrows indicated vaginal epithelial cells, and the red arrows indicated PBC. Black dotted box area at Gram stain (400 ×) was further magnified to Gram stain (1000 ×), which showed the number of countable PBC decreased.

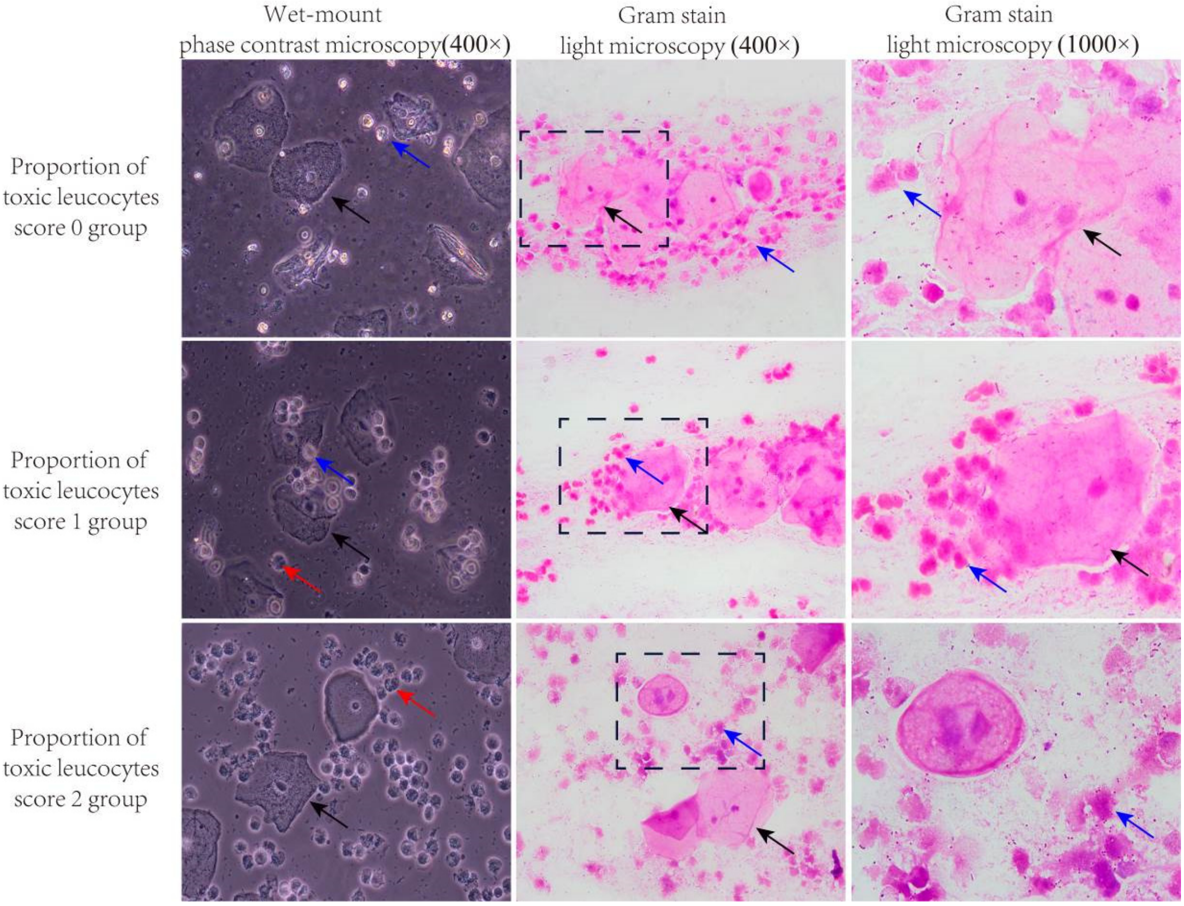

Figure S5. Comparison of proportion of toxic leucocytes between Gram-stained (400×; 1000×) and wet-mount smears (400×). Pictures in the same row from left to right displayed microscopic images at wet mount (400×; phase contrast microscope), Gram stain (1000×; optical microscope) and Gram stain (1000×; oil lens) from the same participant. Pictures in the same column from top to bottom showed microscopic images in according to proportion of toxic leucocytes score 0,1 and 2 from three different participants. The black arrows indicated vaginal epithelial cells, the blue arrows indicated leukocytes without toxic particles, and orange arrows indicated leukocytes with toxic particles. Black dotted box area at Gram stain (400 ×) was further magnified to Gram stain (1000 ×). Toxic leucocytes could be identified at wet mount (400×; phase contrast microscope), but toxic leucocytes could not be identified at Gram stain.

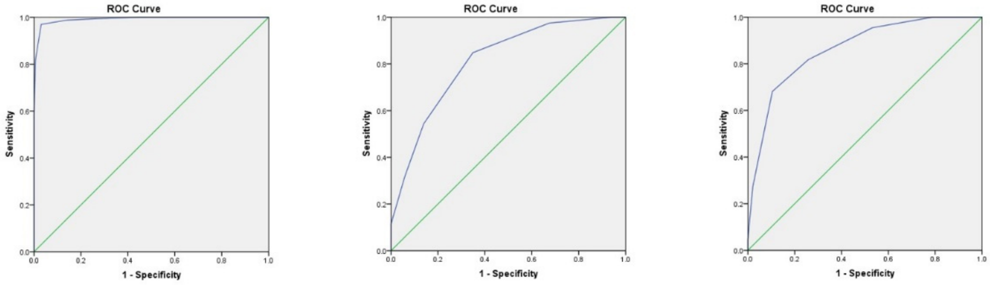

Mild AV Moderate AV Severe AV

Figure S6. The ROC curve for the diagnosis of AV with different levels of severity with new diagnostic criteria
