# Supplementary material for: Aerobic Vaginitis Diagnosis Criteria Combining Gram Stain with Clinical Features: An Establishment and Prospective Validation Study
Source: Diagnostics (Basel). 2022 Jan 13;12(1):185. doi: 10.3390/diagnostics12010185 (PMC8775230; doi:10.3390/diagnostics12010185)
Supplement: Supplementary file 1 [file diagnostics-12-00185-s001.zip › supplementary tables S1-S4.pdf]

Table S1. AV wet-mount microscopy diagnostic score

Score	LBG	Background flora	No. of leucocytes	Proportion of toxic leucocytes	Proportion of PBC
0	Ⅰ, Ⅱa	Unremarkable or cytolysis	≤10/hpf	None or sporadic	None or <1%
1	Ⅱb	Small coliform bacilli	>10/hpf and ≤10/epithelial cell	≤50% of leucocytes	≥1% and ≤10%
2	Ⅲ	Cocci or chains	>10/epithelial cell	>50% of leucocytes	>10%

*LBG: lactobacillary grade, I: predominantly lactobacillary morphological types. IIa: predominantly lactobacilli but mixed with other bacteria. IIb: other bacteria overgrowth but limited numbers of lactobacilli are still present. III: microflora consists of numerous other bacteria, with no lactobacilli present. PBC: parabasal epitheliocytes.

Table S2. The characteristics of participants

	Criteria development population		Validation population
**Characteristics**	**AV (n=325)**	**control (n=325)**	**(n=500)**
Age, years			
Mean±SD	32.99±7.45	31.78±7.14	34.83±9.23
Marital status, n(%)			
Married	240 (73.85%)	305 (93.85%)	418 (83.60%)
Unmarried	85 (26.15%)	20 (6.15%)	82 (16.40%)
Education, n(%)			
College or above	276 (84.92%)	303 (93.23%)	443 (88.60%)
High school or less	49 (15.08%)	22 (6.77%)	57 (11.40%)

Table S3. The accuracy of different clinical features combinations

Different combinations of clinical features	Sensitivity(%)	Specificity(%)	Yorden index
1 score for each of 3 clinical features, total score was 11	96.9	97.0	0.939
pH>4.5: score 1, vaginal hyperemia/yellow discharge: score 1 , total score was 10	96.9	97.5	0.944
Vaginal hyperemia: score 1, pH>4.5/yellow discharge: score 1, total score was 10	96.7	97.5	0.942
Yellow discharge : score 1, vaginal hyperemia /pH>4.5: score 1, total score was 10	96.3	97.9	0.942

1 score for pH>4.5/vaginal hyperemia/yellow discharge, total score was 9	98.8	88.5	0.873

Table S4. Diagnostic performance of new criteria for diagnosing AV

	**AV**		**Different severity of AV***			**Sensitivity (%)**	**Specificity (%)**	**Youden index**	**Four classification accuracy# (%)**
	**Control**	**AV**	**Mild AV**	**Moderate AV**	**Severe AV**				
Wet-mount	325	325	172	118	35	96.92	97.54	0.944	84.92
New criteria	317	315	128	83	24				

*AV severity: Wet-mount Mild AV 3～4, Moderate AV 5～6, Severe AV 7～10；

New criteria Mild AV 4～5, Moderate AV 6～7, Severe AV 8～10；

Four classification accuracy: The accuracy of new criteria for diagnosing normal and mild, moderate and severe AV
